# Cellulose and Cellulose Synthase in a Marine *Pseudomonas* Strain from Antarctica: Characterization, Adaptive Implications, and Biotechnological Potential

**DOI:** 10.3390/md23100410

**Published:** 2025-10-21

**Authors:** Maria Chiara Biondini, Martina Di Sessa, Alberto Vassallo, Federica Chiappori, Marco Zannotti, Alessio Mancini, Rita Giovannetti, Sandra Pucciarelli

**Affiliations:** 1 School for Advanced Studies, Istituto Universitario di Studi Superiori (IUSS), 27100 Pavia, Italy; mariachiara.biondini@unicam.it (M.C.B.); martina.disessa@unicam.it (M.D.S.); 2 School of Biosciences and Veterinary Medicine, University of Camerino, 62032 Camerino, Italy; alberto.vassallo@unicam.it (A.V.); alessio.mancini@unicam.it (A.M.); sandra.pucciarelli@unicam.it (S.P.); 3 School of Science and Technology, Chemistry Division, Chemistry Interdisciplinary Project (ChIP), University of Camerino, 62032 Camerino, Italy; rita.giovannetti@unicam.it; 4 Consiglio Nazionale delle Ricerche—Istituto di Tecnologie Biomediche CNR-ITB, 20054 Segrate, Italy; federica.chiappori@itb.cnr.it; 5 IridES S.r.l., Via Via Gentile III da Varano n° 1, 62032 Camerino, Italy

**Keywords:** biopolymers, green protocols, deep learning tools, molecular modeling

## Abstract

Antarctic microorganisms have developed extraordinary strategies for adaptation. They have also demonstrated the ability to produce various biopolymers in response to environmental stress. The demand for biopolymers is constantly increasing and is expected to grow further. Among emerging biomaterials, bacterial cellulose (BC) is generating significant interest due to its unique characteristics that distinguish it from plant-based cellulose. BC exhibits higher purity, water-holding capacity, and tensile strength compared to its plant-based counterpart. Furthermore, BC can be obtained through environmentally friendly protocols. Several bacterial strains have already been identified as cellulose producers, including *Komagataeibacter xylinus*. In this study, a marine bacterial strain named *Pseudomonas* sp. ef1, isolated from a consortium associated with the Antarctic ciliate *Euplotes focardii*, was tested for cellulose production. We found that this Antarctic *Pseudomonas* can produce BC in conditions that appear unique to this bacterial strain. Furthermore, the final BC product is structurally different from that obtained from the well-known BC producer *Komagataeibacter xylinus*. Additionally, a putative cellulose synthase was identified from the *Pseudomonas* sp. ef1 genome, exhibiting unique characteristics that may account for the unique BC production capability of this Antarctic marine *Pseudomonas*. The versatility of BC opens numerous applications, including in papermaking, food, pharmaceutical, and biomedical sectors.

## 1. Introduction

Antarctica provides a unique natural laboratory to investigate the evolutionary processes behind environmental adaptation. Antarctic microorganisms have developed extraordinary survival strategies, including the ability to resist cold temperatures, oxidative stress, and UV radiation, to scavenge iron present in limited concentrations, and to detoxify hazardous compounds, such as heavy metals and pollutants [1]. They have also shown the ability to produce different pigments and biopolymers in response to environmental stress factors [1]. Cellulose forms the basic structural foundation of the primary cell wall of green plants, algae, and fungi [2] and it is the main constituent of natural fibers such as cotton [3]. It is a polysaccharide consisting of a linear chain of several β (1 → 4) linked D-glucose units and represents the most abundant organic polymer on Earth [3]. Cellulose is also present in bacteria, the so-called nanocellulose [4,5]. The biosynthesis of bacterial cellulose (BC) has been observed many years ago by ancient Chinese growing the Kombucha tea mushroom, a syntrophic colony of acetic acid bacteria and yeast, which metabolizes sugar to produce a slightly acidic tea-colored drink and forms a thick cellulosic mat at its surface [6]. BC was then reported by Brown in 1886, who identified the growth of a non-branched pellicle with a structure chemically equivalent to that of plant cellulose [7].

In recent years, BC has been evaluated as a promising polymer for biotechnological application. For example, it can be used in the food industry, serving as a novel biological material and edible packaging [8]. In the medical field, BC finds use as a wound dressing material, artificial skin, vascular grafts, scaffolds for tissue engineering, artificial blood vessels, wound pads, and dental implants. Furthermore, BC has industrial applications, such as acting as a sponge to collect leaking oil and as a material for absorbing toxins [9]. In both plants and bacteria, cellulose is synthesized by cellulose synthase enzymes (CesAs). This complex varies considerably by kingdom; however, it shares a conserved catalytic subunit termed BcsA (bacterial cellulose synthase subunit A) in prokaryotes and CesA in eukaryotes [10]. Plants’ CesA was derived from bacterial cellulose synthase upon the endosymbiosis event that led to the formation of chloroplasts [11]. Very few bacterial species can synthesize BC, and they include *Komagataeibacter xylinus* (previously known as *Gluconacetobacter xylinus*), some *Agrobacterium* spp., *Azotobacter*, *Rhizobium* spp., *Sarcina*, *Alcaligenes*, and *Pseudomonas* genera [4]. *K. xylinus* is the most studied and the most efficient BC producer [12]. It is an aerobic Gram-negative bacterium with fermentation activity in the pH range of 3–7 and a growth temperature range of 25–30 °C, using saccharides as a carbon source [13]. *K. xylinus* can use various sugars and produces relatively high yields of cellulose in liquid medium [14,15].

In the present study, we report the biosynthesis of BC from a marine strain isolated from a bacterial consortium associated with the Antarctic ciliate *Euplotes focardii* [16,17] and named *Pseudomonas* sp. ef1. *E. focardii* is a free-swimming ciliate, endemic to the oligotrophic coastal sediments of Terra Nova Bay, and is classified as an obligatory psychrophilic stenothermal organism [18,19,20,21]. *Pseudomonas* sp. ef1 was previously demonstrated to be able to transform heavy metals such as copper, nickel, and silver into nanoparticles showing antibiotic activity [22,23,24], and to produce unique pyoverdines [25]. This strain can produce BC using synthesis media conditions that are unique. BC materials have been chemically characterized and compared with that obtained from the most efficient BC producer *K. xylinus*. *Pseudomonas* BC was synthesized with different shapes and structural characteristics as visualized under SEM analysis. Additionally, a putative *Pseudomonas* sp. ef1 cellulose synthase was identified and characterized.

## 2. Results

### 2.1. BC Production

**Figure 1:**
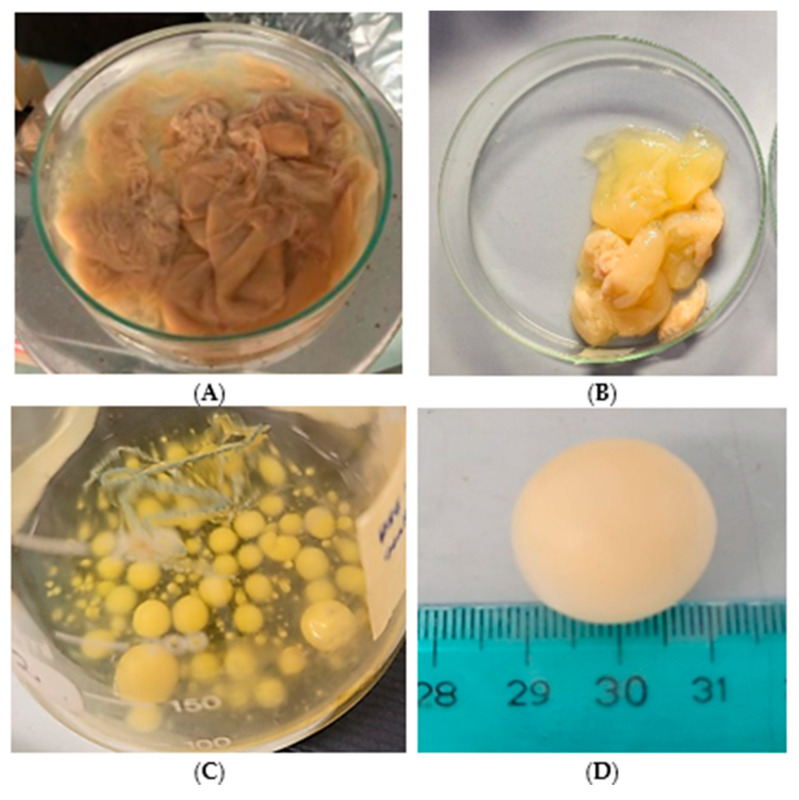
Cellulose produced by *Pseudomonas* sp. ef1 (**A**) and *K. xylinus* (**B**) in HS medium under static conditions, and cellulose synthesized by *Pseudomonas* sp. ef1 in artificial marine water under agitation (**C**,**D**).

The capacity of *Pseudomonas* sp. ef1 to synthesize BC through fermentation was evaluated in standard HS medium containing 1.5% glucose, which is commonly utilized for *K. xylinus*, the most well-known BC producer. BC production by *Pseudomonas* sp. ef1 was assessed in static and shaking conditions. Under static conditions, the BC produced by *Pseudomonas* sp. ef1 exhibited a sheet-like form and was more dispersed in water (Figure 1A), differing from the BC produced by *K. xylinus*, which had a more gelatinous appearance (Figure 1B). *Pseudomonas* sp. ef1 can generate BC at a pH 6.5 and temperatures ranging from 22 to 24 °C. In contrast, the optimal conditions for BC production in *K. xylinus* are at pH 6 and temperatures between 28 and 30 °C. No BC production was detected under shaking conditions.

Since *Pseudomonas* sp. ef1 is a marine bacterium, BC production was also examined in artificial seawater supplemented with 1.5% glucose at 22–24 °C, both in static and shaking conditions. Cultures were started from those grown in yeast extract (1%) or nutrient broth liquid medium (as described in the Material and Methods). After 5–6 days of incubation under shaking at 100 rpm, the formation of spherical flocculates was observed (Figure 1C,D), with sizes ranging from 3 to 20 mm in diameter. Under these media conditions, BC production under static incubation was not observed. The water content was estimated to be 79%.

To evaluate how cultivation conditions affect BC production, the ratio of starting glucose (in grams) to producing cellulose (in grams) was calculated. The results showed a consistent production efficiency of approximately 30% across all conditions.

### 2.2. Fourier-Transform Infrared (FTIR) Spectroscopic Characterization of BC

**Figure 2:**
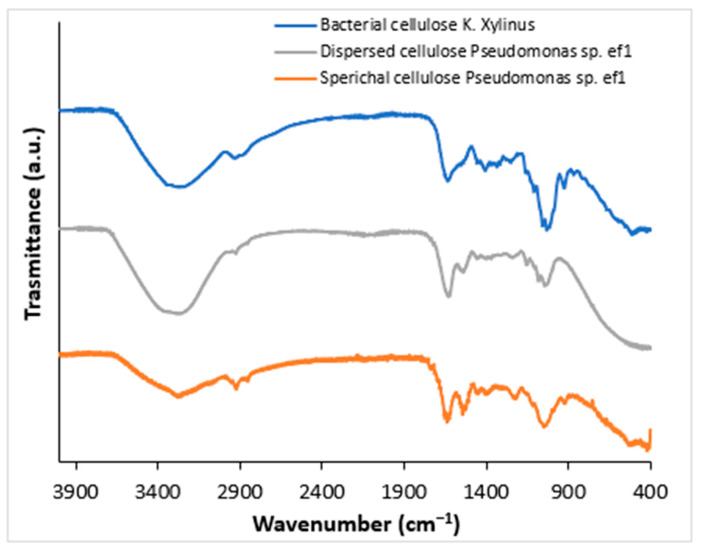
FTIR spectra of bacterial cellulose (BC) produced by *K. xylinus* (blue line) and *Pseudomonas* sp. ef1 (gray and orange lines). The gray line represents the spectrum of sheet-like BC, while orange line corresponds to spherical-shaped BC.

To confirm that the obtained products correspond to BC, Fourier-Transform Infrared Spectroscopy (FTIR) analysis was performed. FTIR spectra were acquired from solid samples after drying both the spherical BC (Figure 2, gray line) and the dispersed sheet-like BC (Figure 2, orange line) and compared to cellulose produced by *K*. *xylinus* (Figure 2, blue line). Both cellulose types, spherical and dispersed, exhibited IR spectra comparable to those of *K*. *xylinus* and standard plant-derived cellulose, as reported by Abderrahim et al. [26]. The broad adsorption band at 3330 cm cm^−1^ is attributed to the -OH stretching, the vibrations in the range 2850–2940 cm^−1^ are relative to the C-H stretching in the bacterial cellulose structure. The peak at around 1600 cm^−1^ can be attributed to the residual water, present in the cellulose network. Several bands have been detected at 1460, 1390, 1320, and 930 cm^−1^ attributed to C−H stretching of CH_2_ and CH_3_ groups, that could indicate the possibility of a methylated cellulose. Furthermore, a strong band is visible at around 1050 cm^−1^ that corresponds to C-O-C and C-O-H vibrations [27,28,29].

### 2.3. Scanning Electron Microscope (SEM) Analysis of Bacterial Cellulose

**Figure 3:**
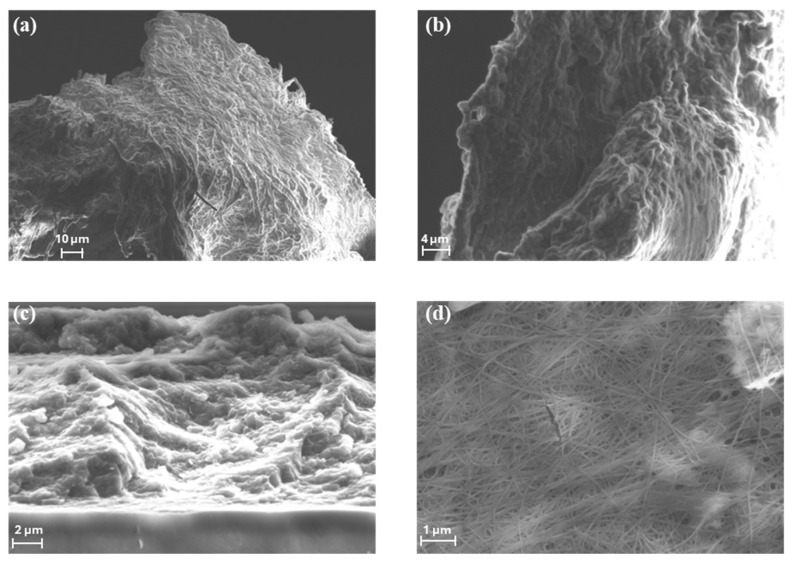
SEM images of (**a**,**b**) the spherical shaped BC from *Pseudomonas* sp. ef1, (**c**) dispersed sheet-like BC from *Pseudomonas* sp. ef1. and (**d**) BC produced by *K. xylinus*.

The morphologies of the BC produced by *Pseudomonas* sp. ef1 and *K. xylinus* samples were evaluated by FE-SEM measurements (Figure 3).

SEM images reveal that the spherical BC produced by *Pseudomonas* sp. ef1 (Figure 3a,b) consists of filamentous structures with diameters below 1 µm, similar to those observed in BC synthesized by *K. xylinus* (Figure 3d). However, the fibrous network of *Pseudomonas* sp. ef1 appears less uniform and less distinctly organized compared to the well-defined and homogeneous architecture of *K. xylinus*-derived BC.

In contrast, the sheet-like BC morphology observed in *Pseudomonas* sp. ef1 (Figure 3c) presents a disordered and loosely connected structure. This irregular arrangement may contribute to its enhanced dispersibility in aqueous environments, suggesting potential advantages for applications requiring high water solubility or colloidal stability.

### 2.4. Powder X-Ray Diffraction (XRD) Analysis

**Figure 4:**
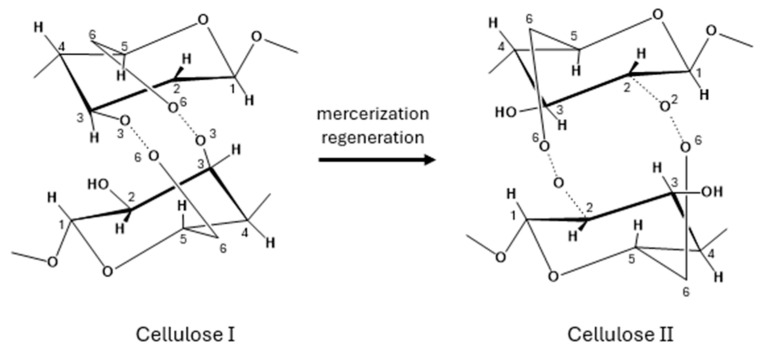
Conversion of Cellulose I to Cellulose II.

XRD was then used to analyze the crystal structure of BC samples. Various polymorphic forms of cellulose are possible; cellulose Iα is typically synthesized by microorganisms, while cellulose Iβ, which is the predominant form, is found in higher plants [30]. Despite both cellulose Iα and Iβ consisting of parallel molecular chains, they exhibit distinct crystal lattice structures: Iα is triclinic, whereas Iβ adopts a monoclinic configuration [31]. Cellulose II can be obtained from either cellulose Iα or Iβ through specific treatments: alkali treatment (mercerization) or dissolution followed by recrystallization (regeneration), respectively (Figure 4).

In some cases, cellulose II has also been found in nature [32,33,34]; unlike cellulose I, which features parallel chains, cellulose II is characterized by an antiparallel chain arrangement and a monoclinic crystal lattice [31].

**Figure 5:**
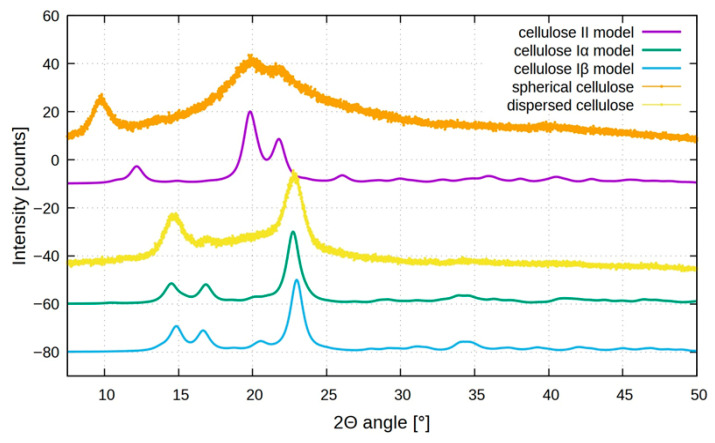
X-ray diffraction patterns of dispersed (yellow) and spherical (orange) bacterial cellulose produced by *Pseudomonas* sp. ef1 in static and agitation conditions, respectively, compared with cellulose Iα (green), cellulose Iβ (blue light), and cellulose II models (violet).

Figure 5 shows the XRD diffractograms of the different BCs. In the XRD spectra, the standard model of cellulose II, Iα, and Iβ are also reported. The diffraction pattern for dispersed sheet-like cellulose (yellow plot) shows three distinct peaks at 2θ = 14.6°, 16.6°, and 22.6°, in agreement with cellulose I structure, as also reported for cellulose produced by *K. xylinus* [35]. These peaks are assigned to the (1 0 0), (0 1 0), and (1 1 0) planes of cellulose Iα or the (1 1 0), (1 1 0), and (2 0 0) planes of cellulose Iβ [36,37]. Usually, cellulose produced by microorganisms is Iα, but distinguishing between the two allomorphs based solely on XRD peak positions is challenging due to their close proximity.

Analyzing the spherical BC, the XRD pattern (orange plot) is quite different, showing distinct peaks at 2θ around 9.5°, 19.5°, and 21.5° that could be assigned to (1-1 0), (1 1 0), and (0 2 0) planes of the cellulose II structure, which is more thermodynamically stable but less ordered compared to cellulose I [38,39,40].

The formation of cellulose II mediated by bacteria under agitation may be attributed to localized thermal effects induced by mechanical stirring. This shift confirms a rearrangement in the cellulose crystalline lattice due to agitation-induced structural changes; agitation not only enhances oxygen and nutrient distribution, but also generates mild heat and mechanical stress, which can promote partial rearrangement of glucan chains with the formation of disordered microdomains or transitional phases. These structural irregularities, including the presence of other compounds, such as bacterial metabolites or salts (high concentration in seawater), could be manifested as atypical reflections in the XRD profile, including the signal below 10°.

### 2.5. Differential Scanning Calorimetry (DSC)

DSC analyses were performed to thermically characterize these bacterial celluloses. As can be seen in Figure S1, during the increase in temperature from 20 to 300 °C, the decrease in the weight takes place in the two samples at about 100 °C, according to reference [41]. This process corresponds to loss of water from the cellulose samples; during the successive temperature increase, further loss of sample mass cannot be observed in the two samples indicating a thermal stability up to 300 °C.

### 2.6. Identification of the Cellulose Synthase and Structural Prediction

To identify the enzyme(s) involved in *Pseudomonas* sp. ef1 BC synthesis, a TBLASTN (2.2.28+) search was conducted on the corresponding genome using the BC synthase operon protein sequences from *K. xylinus* as the query. The accession numbers of the query sequences are listed in Table S1. Only the *K. xylinus* cellulose synthase catalytic subunit (acc. # AHI24410.1), putative cellulose synthase 2 (acc. # AHI24410.1), and cellulose synthase 2 (AHI26282.1) showed a confident match with a single sequence in the *Pseudomonas* sp. ef1 genome in TBLASTN result that correspond to the catalytic subunit A. The other sequences from the *K. xylinus* operon did not show any confident matches. These findings are summarized in Table S1. Therefore, attention was focused on the characterization of this sequence.

Blast search analysis revealed that the identified protein is composed of two different domains: the Scw11 superfamily, which includes the Exo-beta-1,3-glucanase family, and the BcsA superfamily, which includes cellulose synthase catalytic subunit A (Figure S2). The prediction has an E-value of 4 × 10^−17^, indicating a highly reliable result. Therefore, we named the protein putative cellulose synthase catalytic subunit A (hereafter called pBCSA) from *Pseudomonas* sp. ef1.

**Figure 6:**
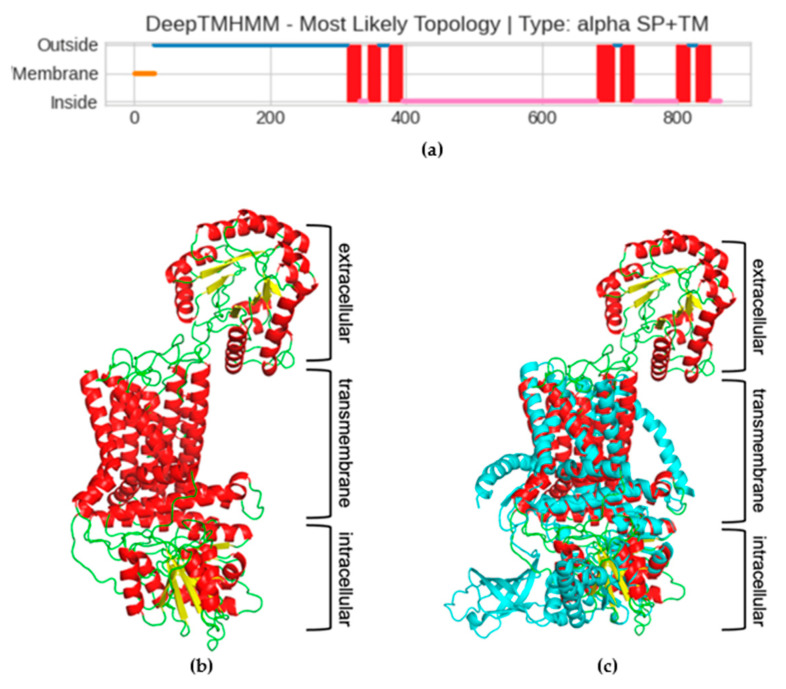
(**a**) Prediction of transmembrane regions of the putative *Pseudomonas* sp. ef1 cellulose synthase subunit A (pBCSA). The plot obtained with DeepTMHMM shows a signal peptide in orange, in blue and pink the extra–intracellular portion, respectively, and in red the transmembrane helixes. (**b**) *Pseudomonas* sp. ef1 pBCSA three-D model, colored according to the secondary structures: helixes in red, strand in yellow, and loop in green. (**c**) Superposed *Pseudomonas* sp. ef1 pBCSA and *C. sphaeroides* cellulose synthase subunit A model, the latter all colored in cyan.

Since the cellulose synthase is known to be a transmembrane (TM) protein, the next step in characterizing the *Pseudomonas* sp. ef1 pBCSA involved predicting the topology of the TM regions. This prediction was carried out using DeepTMHMM, a deep learning model designed to calculate the likelihood of each residue being part of the extracellular, transmembrane, or intracellular region [42]. According to the prediction, a long extracellular domain spanning the first 300 residues (blue line, Figure 6a), three TM-helixes spanning residues from 300 to 400 (red squares in Figure 6a), a long intracellular domain (pink line in Figure 6a), and an additional four TM-helixes at the C-terminal domain were identified. The prediction of the long extracellular domain in the N-terminus was unexpected. To confirm the presence of this domain, the prediction of the 3D structure was carried out using the RoseTTaFold deep learning tool. The model shows very low error for most residues, with the median error at 1.01 Å, as shown in the Figure S3, Error vs. Residue Plot.

Given the constraints on residue length for the protein 3D modelling capabilities of this tool, the *Pseudomonas* sp. ef1 pBCSA amino acid sequence was segmented into three distinct overlapping sections based on transmembrane region predictions. These segments are defined as residues 1–350, 300–490, and 420–860, corresponding respectively to the extracellular domain, the initial three transmembrane helices, and the intracellular domain linked to the final four transmembrane helices. The final 3D model is reported in Figure 6b: the modelling confirmed the presence of the additional extracellular domain, not usually present in most of the BCS subunit A. The presence of this additional domain is even more evident in the superposition of the model with cellulose synthase A subunit of *Cereibacter sphaeroides* [43], which has been used as model template (5EJZ(MMDB) in iCn3D), reported in Figure 6c.

Some strains of *K. xylinus* possess operons which encode a single long BcsAB fusion protein. By contrast, the extracellular domain of the putative *Pseudomonas* sp. ef1 pBCSA corresponds to the Exo-beta-1,3-glucanase family. These enzymes are known to play a key role in the degradation of beta-1,3-glucans, which are polysaccharides found in the cell walls of fungi, some bacteria, and plants. However, these proteins have also a role in biofilm formation and modification (see Section 3).

## 3. Discussion

Cellulose is the key component of plant cell walls and the most abundant biopolymer on Earth [43,44]. While most cellulose is produced by plant cellulose synthase complexes, this enzyme clearly has a bacterial origin: there is no doubt that its genes have been acquired by plants from cyanobacterial ancestors of their chloroplasts [45]. A marine Antarctic *Pseudomonas* strain capable of producing bacterial cellulose from glucose under energy-safe conditions was isolated. We isolated a marine Antarctic *Pseudomonas* strain able to produce BC from glucose, in energy-safe conditions. The produced BC possesses different morphology, according to the protocol used. In HS medium, the produced BC appears as a sheet-like product, whereas in medium containing either yeast extract or nutrient broth and sea water under shaking conditions, the product appears as spherical flocculates.

*K. xylinus* cultures grown in liquid media are remarkably efficient at producing a surface pellicle composed entirely of pure cellulose fibers [4]. The cellulose biosynthesis process in this bacterium is controlled by a four-gene *bcsABCD* operon. Among the corresponding proteins, BcsA and BcsB are essential for in vitro cellulose-synthesizing (BCS) activity. However, all four proteins—BcsA, BcsB, BcsC, and BcsD—are necessary to achieve optimal cellulose production in vivo. This suggests that BcsC and BcsD play critical roles in exporting glucan chains and organizing them into fibers at the cell surface. Certain strains of *K. xylinus* also possess a second *bcs* operon, which encodes a single, elongated BcsAB fusion protein, along with two additional genes, *bcsX* and *bcsY*, whose functions remain uncharacterized [46].

Genomic data revealed unexpected diversity of cellulose synthase operons even in closely related bacteria, indicating substantial differences in cellulose secretion mechanisms [45]. A putative cellulose synthase subunit A was identified in *Pseudomonas* sp. ef1, possessing an extracellular domain represented by a member of the Exo-beta-1,3-glucanase family, differently from some *K. xylinus* strains that possess a single long BcsAB fusion protein. The Exo-beta-1,3-glucanase family are enzymes that play a key role in the degradation of beta-1,3-glucans, which are polysaccharides found in the cell walls of fungi, some bacteria, and plants. However, these proteins have additional biological roles, including antifungal defense mechanisms by degrading fungal cell walls, or in biofilm formation and modulation. The unusual structure of the putative BCS A subunit may account for the sheet-like and water-soluble cellulose organization that is obtained by incubating *Pseudomonas* sp. ef1in HS medium at pH 6.5. This BCS A subunit structure is shared also by other cellulose-producing *Pseudomonas* strains. However, these strains also possess a standard operon organization. By contrast, *Pseudomonas* sp. ef1 appears to have lost the standard operon organization, likely following its adaptation to the Antarctic host environment. The water-soluble form of the *Pseudomonas* ef1 BC makes it particularly well-suited for coating applications, especially in food packaging, as it eliminates the need for additional processing steps—such as homogenization—before spreading it onto other materials.

Bacterial synthesis of cellulose is seen as a convenient and effective way to produce stable recyclable fibers for use in wound dressing and in a variety of emerging nanotechnologies. Furthermore, BC has industrial applications, such as acting as sponges to collect leaking oil and as materials for absorbing toxins [9]. Exploring new methods for cellulose synthesis, beyond traditional vegetable sources, will aid in the development of innovative and renewable materials.

## 4. Materials and Methods

### 4.1. Strains Culturing and Genome Sequencing

DNA used for sequencing was extracted from a culture of *Pseudomonas* sp. ef1 grown overnight in 10 mL of LB (10 g/L tryptone, 5 g/L yeast extract, 10 g/L NaCl) at 23 °C under shaking. Cells were harvested by centrifugation and DNA was extracted by using the ‘DNeasy PowerSoil Pro Kit’ (Qiagen, Milan, Italy), following the protocol provided by the manufacturer. Genomic DNA of *Pseudomonas* sp. ef1 was sequenced with nanopore technology and using a native barcoding approach (since genomic DNA of *Pseudomonas* sp. ef1 was sequenced with other non-related DNA samples). Sequencing was carried out using a MinION Mk1B and a R9.4 flow cell (Oxford Nanopore Technology, ONTO (Oxford, UK). Basecalling was performed with MinKNOW (v24.02.16) using the super accurate model. Sequencing reads were assembled using EPI2ME (v5.1.14) and the workflow ‘wf-bacterial-genomes’ (v1.2.0) provided by ONT (GenBank acc. N. VAUR00000000). Mash’s (v3.1.3) output results (see complementary Material and Methods) suggest that *Pseudomonas* ef1 belongs to the same species as *Pseudomonas triticicola*.

### 4.2. BC Production and Purification

BC production was conducted in Hestrin–Schramm (HS) medium (20 g/L glucose, 5 g/L yeast extract, 5 g/L peptone, 2.7 g/L disodium hydrogen phosphate, 1.15 g/L citric acid) at pH 6.5, under both static and shaking conditions for 3–5 days, at temperatures of 22–24 °C. Alternatively, *Pseudomonas* sp. ef1 BC was produced in artificial seawater (22 ‰) supplemented with 1.5% (*w*/*v*) glucose at 22–24 °C, both in static and shaking conditions, starting from cultures grown in yeast extract (1% *w*/*v*) or nutrient broth liquid medium. *K. xylinus* BC production was conducted in HS medium at pH 6 and temperatures of 28–30 °C. The inoculum consisted of *Pseudomonas* sp. ef1 cells obtained from LB agar plates. The resulting biofilm was collected using a filter, washed multiple times with sterile deionized water, and incubated at 80 °C for 4 h while soaked in sterile deionized water to completely clean BC from bacteria. To evaluate how cultivation conditions affect BC production, the ratio of starting glucose (in grams) to producing cellulose (in grams) was calculated.

### 4.3. BC Characterization

Functional group identification of the bacterial cellulose material was characterized by FT-IR analysis using a Perkin–Elmer System 2000 spectrometer (Waltham, MA, USA) equipped with Pike GladiATR technology. Prior to the analysis, the samples were dried in an oven at 40 °C.

Surface morphology and microstructural features of the dried bacterial cellulose samples, coated with a thin layer of chromium, were examined using a Sigma FE-SEM (Zeiss, Jena, Germany), operated at 5–15 kV.

X-ray diffractometry (XRD) patterns of the samples were recorded using a Bruker D6 powder diffractometer equipped with a Cu source (kα1 = 1.54060 Å) and Lynxeye SSD 160-2 detector (Bruker, Billerica, MA, USA). The bacterial cellulose was dried and ground to a powder before analysis. The samples were scanned over a 2θ range between 5 and 50° and a θ range between 2.5 and 25° for 15 min, with each step recorded at 0.2 s intervals. Generator voltage and filament emission were set to 40 kV and 15 mA, respectively. All the results were compared with the respective models of celluloses I and cellulose II [47,48,49].

The thermal behavior was determined by differential scanning calorimetry (DSC). Samples (2–4 mg in an aluminum concave pan with pierced lid) were analyzed in a DSC 250 TA (TA Instrument DSC 250 TA (TA Instrument, Dover, DE, USA), according to the following thermal program: heating from 25 °C to 300 °C (2 min hold), cooling to −25 °C (2 min hold) and heating to 300 °C, all steps at 10 °C/min.

### 4.4. Identification of the Cellulose Synthase Enzymes, Transmembrane (TM) Regions Prediction and Homology Modeling

*Pseudomonas* sp. ef1 putative cellulose synthase was identified by local TBLASTN search in the corresponding genome using the BC synthase operon protein sequences from *K. xylinus* as the query. The accession numbers of the query sequences are listed in Table S1. The prediction of transmembrane (TM) proteins was conducted using DeepTMHMM, a tool that utilizes deep neural networks [42]. To obtain the structural model of cellulose synthase, the corresponding sequence was divided into three regions corresponding to three structural domains: the extracellular (residues 1 to 350), transmembrane (residues 300 to 490), and the intracellular domain (residues 420 to 860). A model for each domain was obtained using RoseTTaFold, the deep learning tool of Rosetta [50]. The resulting models were manually overlapped using PyMol 3.1 (https://www.pymol.org/, accessed on 10 October 2025).

## 5. Conclusions

In this study, the production of biocellulose by the Antarctic bacteria *Pseudomonas* sp. ef1 was investigated. This bacterium, using glucose as carbon source, is capable to produce BC, in different morphologies, depending on the culture protocol: a sheet-like form of BC in Hestrin–Schramm (HS) medium under static conditions, and spherical BC flocculates in media containing yeast extract or nutrient broth with seawater under shaking conditions. The different materials were characterized by FT-IR, XRD, and DSC analysis confirming the presence of bacterial cellulose, and specifically cellulose I for dispersed sheet-like BC and cellulose II for spherical BC, with thermal stability up to 300 °C. Genomic analysis revealed a putative cellulose synthase subunit A with an extracellular domain belonging to the Exo-beta-1,3-glucanase family, which is unusual and likely contributes to the unique organization and water solubility of the BC produced by this strain. This structural peculiarity may be the result of adaptation to the Antarctic environment.

## 6. Patents

The manuscript is related to patents: no. 102020000031769, 102022000018663, 102021000017333.

## Data Availability

*Pseudomonas* sp. ef1 is available at GCA_007293365.1.

## Supplementary Materials

The following supporting information can be downloaded at: https://www.mdpi.com/article/10.3390/md23100410/s1, Figure S1: DSC curves for dispersed and spherical bacterial cellulose produced by *Pseudomonas* sp. ef1 in static and agitation condition, respectively; Figure S2: Blast search result using the putative cellulose synthase A from *Pseudomonas* sp. Ef1.as query; Figure S3: Error vs. Residue Plot. The model shows very low error for most residues, with the median error at 1.01 Å; Table S1: tBlastN results of the *Pseudomonas* sp. Ef1 genome analysis to search for BC synthesis genes using as query the BC synthesis operon sequence from *Komagataeibacter xylinus* E25 strain.
