# Supplementary material for: Cellulose and Cellulose Synthase in a Marine Pseudomonas Strain from Antarctica: Characterization, Adaptive Implications, and Biotechnological Potential
Source: Mar Drugs. 2025 Oct 21;23(10):410. doi: 10.3390/md23100410 (PMC12565738; doi:10.3390/md23100410)
Supplement: Supplementary file 1 [file marinedrugs-23-00410-s001.zip › marinedrugs-3890592-supplementary.pdf]

Cellulose and cellulose synthase in a marine *Pseudomonas* strain from Antarctica: Characterization, Adaptive Implications, and Biotechnological Potential.

Maria Chiara Biondini [1,2], Martina Di Sessa [1,3], Alberto Vassallo [2], Federica Chiappori [4], Marco Zannotti *[3,5], Alessio Mancini[2,5], Rita Giovannetti [3,5] and Sandra Pucciarelli [2,5]

1 School for Advanced Studies, Istituto Universitario di Studi Superiori (IUSS), 27100 Pavia, Italy; mariachiara.biondini@unicam.it (M.C.B.); martina.disessa@unicam.it (M.D.S.)
2 School of Biosciences and Veterinary Medicine, University of Camerino, 62032 Camerino, Italy; alberto.vassallo@unicam.it (A.V.); alessio.mancini@unicam.it (A.M.); sandra.pucciarelli@unicam.it (S.P.)
3 School of Science and Technology, Chemistry Division, Chemistry Interdisciplinary Project (ChIP), University of Camerino, 62032 Camerino, Italy; rita.giovannetti@unicam.it
4 Consiglio Nazionale delle Ricerche—Istituto di Tecnologie Biomediche CNR-ITB, 20054 Segrate, Italy; federica.chiappori@itb.cnr.it
5 IridES S.r.l., Via Via Gentile III da Varano n° 1, 62032 Camerino, Italy
* Correspondence: marco.zannotti@unicam.it

Table S1. tBlastN results of the *Pseudomonas* sp. Ef1 genome analysis to serach for BC synthesis genes using as quary the BC synthesis operon sequence from Komagataeibacter xylinus E25 strain

Query=			Sequences producing significant alignments on *Pseudomonas* sp. Ef1 genome		
Acc number	name	sequence	contig	e-value	Predicted sequence
AHI24410.1	cellulose synthase catalytic subunit	>AHI24410.1 cellulose synthase catalytic subunit [Komagataeibacter xylinus E25] subunit A<br>MSEVQSSAPAESRFDRIS<br>NKILSLRGASYIVGAVGIF<br>ALLAATTVTLSINEQLIV<br>ALICIAVFFIVGRHK<br>SRRTQVFLEVLSALVSLR<br>YLTWRLTETLDFDTWIQ<br>GGLGVTLLLAELYALYM<br>LFLSYFQTISPLHRAPLP<br>LSPNPDDWPTVDIFVPTY<br>DESLGIVRLTVLGALGID<br>WPPDKVNVYILDDGERE<br>EFARFAEECGARYIARP<br>DSAHAKAGNLNYAIKHT<br>TGDHILILDCDHIPTRAFL<br>QIAMGWMVDDPTIALMQ<br>TPHHFYSPDPFQRNLAV<br>GYRTPPEGNLFYGVIQAG<br>NDFWDATFFCGSCAILRR<br>KAIEEIGGFATETVTEDA<br>HTALRMQRKGWSTAYL<br>RIPLASGLATERLVTHIGQ<br>RMRWARGMIQIFRVDNP<br>MMGPGLKLGQRLCYLSA<br>MTSFFFAIPRVVFLASP<br>LAFLFFNQNIIAASFVAVL<br>AYAIPHMFHSIATAAKGN<br>KGWRYSFWSEVYETVM<br>ALFLVRVTIVTLLFPSK<br>GKFNVTEKGGVLEHEEF<br>DLGATYPNIIFACIMALG<br>LLRGGYALIFQHLDIISER<br>AYALNCIWSVISLIIL<br>LAAVAVGRETKQIRQGH<br>RIEAHIPVTVYDYEGNSS<br>HGITEDVSMGGVAIHMP	contig00014	4e-017	>PROKKA_00265 Cellulose synthase 1<br>MSSRKFGLNLVVVLAIAALFTGFWALVNRPVTAPNWPQ<br>QISGFSYSPFQQGQFPQKDQYP<br>SDDEMRRDLEIMSKLTDNIRIYSVDGSLGDIPKLAEEFGLR<br>VTLGIWISPDQERNEREIT<br>KAIELANTSRSVVRVVVGNEALFREEITPEALIVLLDRVR<br>AAVKVPVTTSEQWHIWEKYP<br>QLAKHVDLIAAHVLPYWEFIPVDKAGQFVFDRARDLKKL<br>FPKKPLLLSEVGWPSNGRMRG<br>GADASPADQAIYLRTLVNKLNRQGFNYFVIEAFDQPWKA<br>SDEGSVGAYWGVFNAARQQKF<br>NFEGPVVAIPQWRVLAIGSVVLALLSLTLLMIDGSALRQR<br>GRTFLTFIAFLCGSVLVYIG<br>YDYSQQYSTWFSLTVGFLLALGALGVFIVLLTEAHELAE<br>AVWIHKRRREFLPVLGDSDYR<br>PKVSIHVPCYNEPPEMVKQTLDALAALDYPDFEVLIIDNN<br>TKDPAVWEPVRDYCATLGPR<br>FKFFHVSPLAGFKGGALNYLIPHTAKDAEVIAVIDSDYCV<br>HPNWLKHMVPHFADPKIAVV<br>QSPQDYRDQNESTFKKLCYAEYKGFFHIGMVTRNDRDAI<br>IQHGTMTMTRRSVLEELGWAD<br>WCICEDAELGLRVFEKGLSAAYYHDSYGKGLMPDTFIDF<br>KKQRFRWAYGAIQIIKRHTRS<br>LLRGKDTELTRGQRYHFLAGWLPWVADGMNIFFTVGAL<br>LWSAAMIIVPQRVDPPLLIFAI<br>PPLALFVFKVGKIIFLYRRAVGVNLKDAFCAALAGLALSH<br>TIAKAVLYGFFTTSIPFFRT<br>PKNADNHGFWVAISEAREELFIMLLLWGAALGIFLVQGIP<br>SNDMRFWVTMLLVQSLPYLA<br>ALIMAFLSSLPKPAAKAEPAPVV

		WRDMTPDQPVQVVVHTIL<br>DGEEMNLPATMIRSARG<br>KAVFTWSITNIQVEAAVI<br>RFVFGRADAWLQWNNY<br>EDDRPLRSLWSLILSIKAL<br>FRRKGQMIAQSRPKNKPI<br>ALPVERREPTTSQGGQKQ<br>EGKISRAAS			
AHI24411.1	Cellulose synthase 1	Komagataeibacter xylinus E25		none	
AHI24412.1	Cellulose synthase operon protein C				
AHI24413.1	Cellulose synthase operon protein D				
AHI25956.1	BcsB: Putative cellulose synthase 2 cellulose synthase (UDP-forming) activity	>AHI25956.1 Putative cellulose synthase 2 [Komagataeibacter xylinus E25] A e B<br>MATPRGSKWLVSFLLAG<br>GAFLCLSVGNITLAPEQQ<br>AYISVGTISLFFLLNRRKG<br>RHVTCILMMLSLFVSF<br>RYLIWRLGSTVQFTGPLQ<br>IVMSVALLMAEGYALST<br>LCLSYFQMSWPLGRRPH<br>RLPDNPDEWPMVDVYVPS<br>YNEDLELVRSTVLGAMD<br>LHWPADKLNVYILDDGR<br>RKSFYDFARESGAGYIIR<br>AENNHAKAGNLNHAMKIT<br>RGEFVVIFDCDHVPTRSF<br>LLKTIGWMMADPNLALL<br>QTPHHFYSPDPFQRNLAA<br>GYDVPPEGNMFYGLVQD<br>GNDFWDATFFCGSCAAI<br>RRSALLSVGGFATETVTE<br>DAHTALKMQRKGWGTA<br>YLRQPLAGGLATERLILHI<br>GQRVRWARGMLQIMRL<br>DNPLLGGGLRWEQRLCY<br>LSAMSHFLFAIPRVTFLVS<br>PLGFLFFGQNIIAASPFA<br>IMVYALPHIFHSIMTLSRI<br>EGRWRYSFWSEIYETSLA<br>LFLVRITIVTLLQPHKGKF<br>NVTDKGGLLARGYF<br>DFSAVYPNAIMALVLFV<br>GMVRGILGMIFQYHQKL<br>AFQSFALNTLWITVSLIV<br>VLASIAVGRETRQIRHKP<br>RVRVKLPVDVCFDDGAV<br>FHAHTTDISLGGAGVTLS<br>LPRQLETPLDVTLRYSKP<br>DDEIMVAVPATILGQRG<br>SWLHLQWKIETLEQERQI<br>VSLVFGRSDAWHNWAD<br>FKDDRPLNSIYQVIKSISG<br>LLSPPYLWDLSPAQESE	contig00014	3e-016	>PROKKA_00265 Cellulose synthase 1<br>MSSRKFGLNLVVVLAIAALFTGFWALVNRPVTAPNWPQ<br>QISGFSYSPFQQGQFPQKDQYP<br>SDDEMRRDLEIMSKLTDNIRIYSVDGSLGDIPKLAEEFGLR<br>VTLGIWISPDQERNEREIT<br>KAIELANTSRSVVRVVVGNEALFREEITPEALIVLLDRVR<br>AAVKVPVTTSEQWHIWEKYP<br>QLAKHVDLIAAHVLPYWEFIPVDKAGQFVFDRARDLKKL<br>FPKKPLLLSEVGWPSNGRMRG<br>GADASPADQAIYLRTLVNKLNRQGFNYFVIEAFDQPWKA<br>SDEGSVGAYWGVFNAARQQKF<br>NFEGPVVAIPQWRVLAIGSVVLALLSLTLLMIDGSALRQR<br>GRTFLTFIAFLCGSVLVYIG<br>YDYSQQYSTWFSLTVGFLLALGALGVFIVLLTEAHELAE<br>AVWIHKRRREFLPVLGDSDYR<br>PKVSIHVPCYNEPPEMVKQTLDALAALDYPDFEVLIIDNN<br>TKDPAVWEPVRDYCATLGPR<br>FKFFHVSPLAGFKGGALNYLIPHTAKDAEVIAVIDSDYCV<br>HPNWLKHMVPHFADPKIAVV<br>QSPQDYRDQNESTFKKLCYAEYKGFFHIGMVTRNDRDAI<br>IQHGTMTMTRRSVLEELGWAD<br>WCICEDAELGLRVFEKGLSAAYYHDSYGKGLMPDTFIDF<br>KKQRFRWAYGAIQIIKRHTRS<br>LLRGKDTELTRGQRYHFLAGWLPWVADGMNIFFTVGAL<br>LWSAAMIIVPQRVDPPLLIFAI<br>PPLALFVFKVGKIIFLYRRAVGVNLKDAFCAALAGLALSH<br>TIAKAVLYGFFTTSIPFFRT<br>PKNADNHGFWVAISEAREELFIMLLLWGAALGIFLVQGIP<br>SNDMRFWVTMLLVQSLPYLA<br>ALIMAFLSSLPKPAAKAEPAPVV

		EGDESVHKEETLEKKSLV<br>VPPVHRGIGYGMTMMA<br>AGAGMLLSVVMPAMAQ<br>AQDAAPPAPPAPSQVYTT<br>TG<br>VSRLTPLGDTNSGDIPPV<br>DSMADPTLADRVADTEIT<br>RTIPFHDLGLENGPLTLR<br>GFSPLQGIDVVVPANR<br>VVTHATLKLSGAISPSLLP<br>EATALTVTLNEQYVGTIR<br>IDPAHPTFGPMEFAVDPL<br>YFTGDNKLNFHFAGE<br>YRRDCNDLYNDVLWAQI<br>SDQSSITLTTARIVPERDL<br>SRLPVPFFDSNLKVAMRV<br>PVVMPAAAPGKDIMRA<br>GGLVASWFGKMADSRRL<br>SFPVSRSMPASGNAVEIG<br>QDIPVDDQGHMPGGPTL<br>LEIANPNDKWGTILVVTG<br>RTPHEVEVAARALVFSPD<br>TLGSTASTVVQDVTLRPR<br>RPYDAPAFIPTDRPVRFG<br>ELVTAGDLQAGGFTPG<br>TLHVPFHLPPDLYTWHG<br>LPFLMDLWVRTPDNPVV<br>DISASRLDVSLNNSYLQS<br>YSLLGNSLWRTWSERMV<br>T<br>QHAGAVGHVTALPSWLL<br>FGQNDLQFNFDTRPVDR<br>GACRRTPDNLRLGIDSDS<br>VLDFRRGVHFAMLPNLS<br>Y<br>FSEIGFPFSRMADLGETTL<br>VLPPTPDTDTIGAYLDLM<br>GFMGSVTWYPAAGLHLA<br>TTTDIAHNPPSGDIIL<br>LAPVGQMGPAASLLSRS<br>AYQIDGGHIHVGQDMGL<br>QGIWYMFQDRDGSGLRN<br>GVTANLNAPVHNVAMMI<br>GA<br>ESPYASHRSVLALLGDDG<br>AHIHELVTSLHDRKALSS<br>LQGDLVLKNGDKFTTYR<br>TSPTYTVGSLPLWMWVD<br>WFLSRHPILMYLCGVAG<br>AFMLGTGAWLWLRARA<br>RRRVREQAIADEAARLA<br>GHVPGD			
AHI262<br>77.1	Cellul<br>ose<br>synth<br>ase 2<br>opero<br>n<br>protei<br>n C				
AHI262<br>79.1	Cellul<br>ose<br>synth<br>ase 2<br>opero<br>n<br>protei<br>n C				
AHI262<br>82.1	Cellul<br>ose<br>synth<br>ase 2	>AHI26282.1 Cellulose<br>synthase 2<br>[Komagataeibacter xylinus<br>E25] A e B<br>MTGKVAGLLARTGLDRV<br>PVWVPVVLGVVIMAFVG<br>SVTVDPAMQGFIAIGTIT<br>VLLVLNRRPGRGITIFLM		2e-016	>PROKKA_00265 Cellulose synthase 1<br>MSSRKFGLNLVVVLAIAALFTGFWALVNRPVTAPNWPQ<br>QISGFSYSPFQQGQFPQKDQYP<br>SDDEMRRDLEIMSKLTDNIRIYSVDGSLGDIPKLAEEFGLR<br>VTLGIWISPDQERNEREIT<br>KAIELANTSRSVVRVVVGNEALFREEITPEALIVLLDRVR<br>AAVKVPVTTSEQWHIWEKYP

		MLSLLMSLRYIVWRLTM<br>TVQFNNWLQATLALMLL<br>AAETYALVTLCLSYFQM<br>AWPLRRREHPLPDDTAQ<br>WP<br>SVDVFVPSYNEELSLVRS<br>TVLGALDLDWPEDRLNV<br>YILDDGRRKAFRDFALAS<br>GAGYIVRAENNHAKAGN<br>LNHALQVTHSQFAVIFDC<br>DHVPTRGFLKRTVGWM<br>MADPNLALLQTPHHFYA<br>PDPFQRNLASGMHVPPE<br>GN<br>MFYGLVQDGNDFWDAT<br>FFCGSCAVIRREAVMKIG<br>GFATETVTEDAHTALKM<br>QRRGWGTAYLREPLAAG<br>LS<br>TERLILHIGQRVRWARG<br>MLQIMRLDNPLLGGGLR<br>WEQRLCYLSAMSHFLFA<br>MPRAMFLMSPLAYLFLG<br>QN<br>IIAASPLAITVYALPHIFHS<br>VLTLSRIEGRWRYSFWSE<br>IYETSLALFLIRITIVTLLQ<br>PHKGKFNVTDKG<br>GLLARGYFDFSAVYPNVI<br>LALVLFAAFVRGIFGIFW<br>QFHDRLALQSFVLNTLW<br>VVVSLIIVLASIAVGRE<br>TRQTRHAPRITVQLPMAI<br>TDAHGRRFDGHTHDISLG<br>GLAVKGNWAAPEMEGT<br>EVTVHYDNARDGIHVGV<br>P<br>ARVLGVGQGMLRLQWL<br>RRDLQDERQIVGMVFGR<br>NDAWTNWADFPPDRPLR<br>SLAMVFRSIGGLFGRRGP<br>DA<br>ARAMPDGGEENLPATEE<br>KLEKQSLVLKPVPRSVRN<br>GATASAALLVAFTALVP<br>AALAQEAAPAAMAPVA<br>DQ<br>TGVTTDAPFGDSNTGNIP<br>DAAPVIDPLAAERISDTE<br>VTRTITFRNLGAATGPLT<br>LRGYSPLQGLDVVVPA<br>NRVVTHAQLTLSGALSPS<br>LLPEASAVTVTMNEQYV<br>GTIKVDPQHPQFGPLTFDI<br>DPLYFTGDNKLNFRFA<br>GEYRRDCNDLFNEILWA<br>RISDLSHITLTTVRITPERK<br>LSRLPAPFFDPNLRSTLRV<br>PVVLPVTGDRGALK<br>AAGLLASWFGKVADFRK<br>LSFPVSATIPASGNAVEV<br>GENLAVDADGTRPAGPM<br>VAEIANPNDHWGTVLVV<br>T<br>GRNAQEVEVAARAVVFS<br>PDTLGGVASKVVSDVNL<br>ETRQPYDAPAFVPTDRPV<br>RFGELVGAADLQGGGFT<br>P<br>AGMTLPFHLPPDLYTWR<br>GRPFLMNLWIRAPGGPV<br>VDLETSRVDVSVNNNYL<br>QSYTLSPPGLWRKWSER<br>LV<br>NQHAGAAGHVTALPPWL<br>LFGQNQLQFNFDARPIDR			QLAKHVDLIAAHVLPYWEFIPVDKAGQFVFDRARDLKKL<br>FPKKPLLLSEVGWPSNGRMRG<br>GADASPADQAIYLRTLVNKLNRQGFNYFVIEAFDQPWKA<br>SDEGSVGAYWGVFNAARQQKF<br>NFEGPVVAIPQWRVLAIGSVVLALLSLTLLMIDGSALRQR<br>GRTFLTFIAFLCGSVLVYIG<br>YDYSQQYSTWFSLTVGFLLALGALGVFIVLLTEAHELAE<br>AVWIHKRRREFLPVLGDSDYR<br>PKVSIHVPCYNEPPEMVKQTLDALAALDYPDFEVLIIDNN<br>TKDPAVWEPVRDYCATLGPR<br>FKFFHVSPLAGFKGGALNYLIPHTAKDAEVIAVIDSDYCV<br>HPNWLKHMVPHFADPKIAVV<br>QSPQDYRDQNESTFKKLCYAEYKGFFHIGMVTRNDRDAI<br>IQHGTMTMTRRSVLEELGWAD<br>WCICEDAELGLRVFEKGLSAAYYHDSYGKGLMPDTFIDF<br>KKQRFRWAYGAIQIIKRHTRS<br>LLRGKDTELTRGQRYHFLAGWLPWVADGMNIFFTVGAL<br>LWSAAMIIVPQRVDPPLLIFAI<br>PPLALFVFKVGKIIFLYRRAVGVNLKDAFCAALAGLALSH<br>TIAKAVLYGFFTTSIPFFRT<br>PKNADNHGFWVAISEAREELFIMLLLWGAALGIFLVQGIP<br>SNDMRFWVTMLLVQSLPYLA<br>ALIMAFLSSLPKPAAKAEPAPVV

		GACRRTPGDIRMSVDSDS TLDFRRGHHFAQLPNLS YFAEAAFPFSRMADLSET TVVLPDHPDAGTAGAFL DLMGFFGASTWYPAAGV EIMKADEVEQAPPKGDIV VLGTAAQLGRATAVLAR SPYVIHDQHITVGQHMGL QGIWYLFQDRDHAGLKN GVTANLNAPIAEAGVLL A AQSPYDSQRSVVAFMGD TPERIHDMVLSLRNKTDL PLLQGDLVLKNGDRFTS YRTAPLYTVGTLPLWMR L DWYLGRHPALLYLAGLA GAGLAALGIWAWLRGW SRKRVAHDDLTGDL			

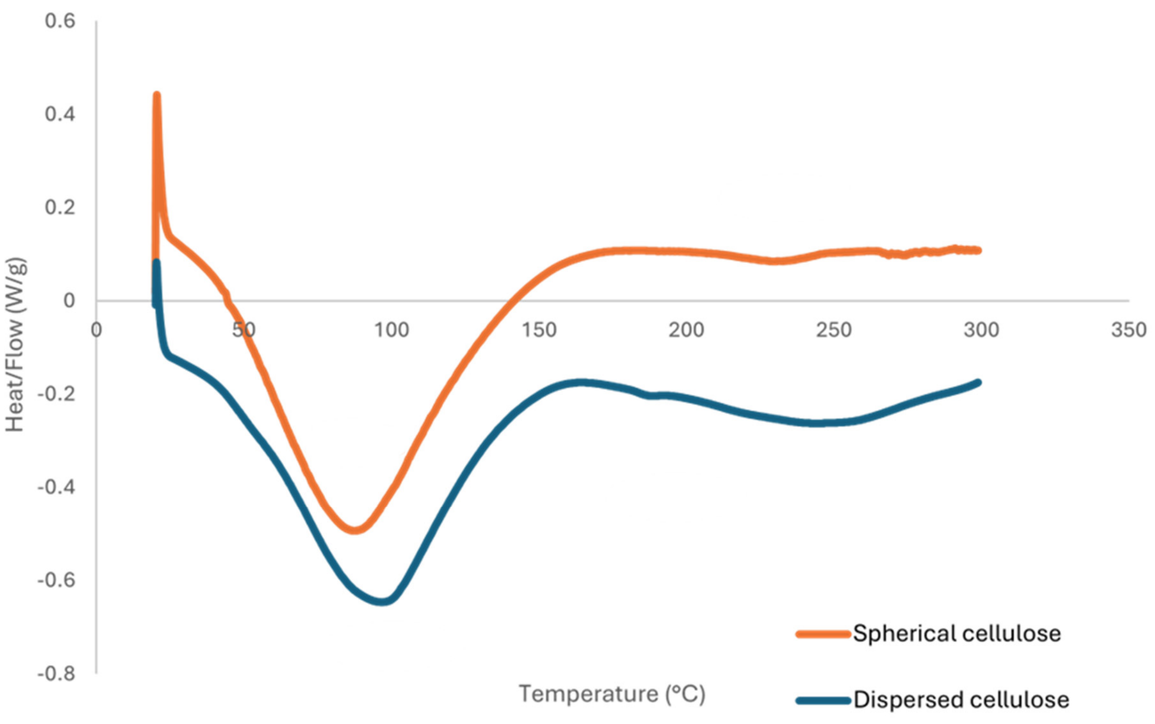

Figure S1. DSC curves for dispersed and spherical bacterial cellulose produced by Pseudomonas sp. ef1 in static and agitation condition, respectively.

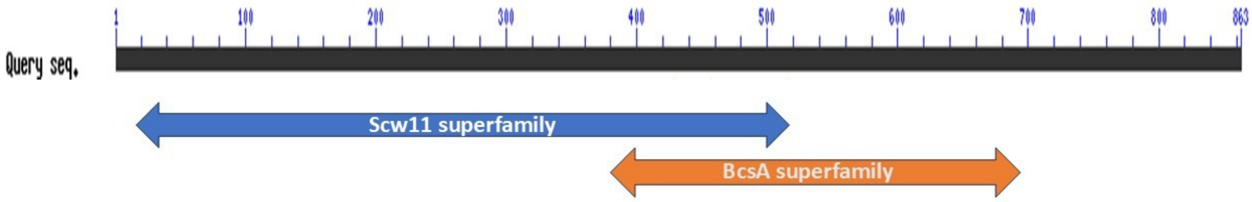

Figure S2. Blast search result using the putative cellulose synthase A from *Pseudomonas* sp. Ef1.as query.

The analysis revealed that the identified protein is composed by two different domains.

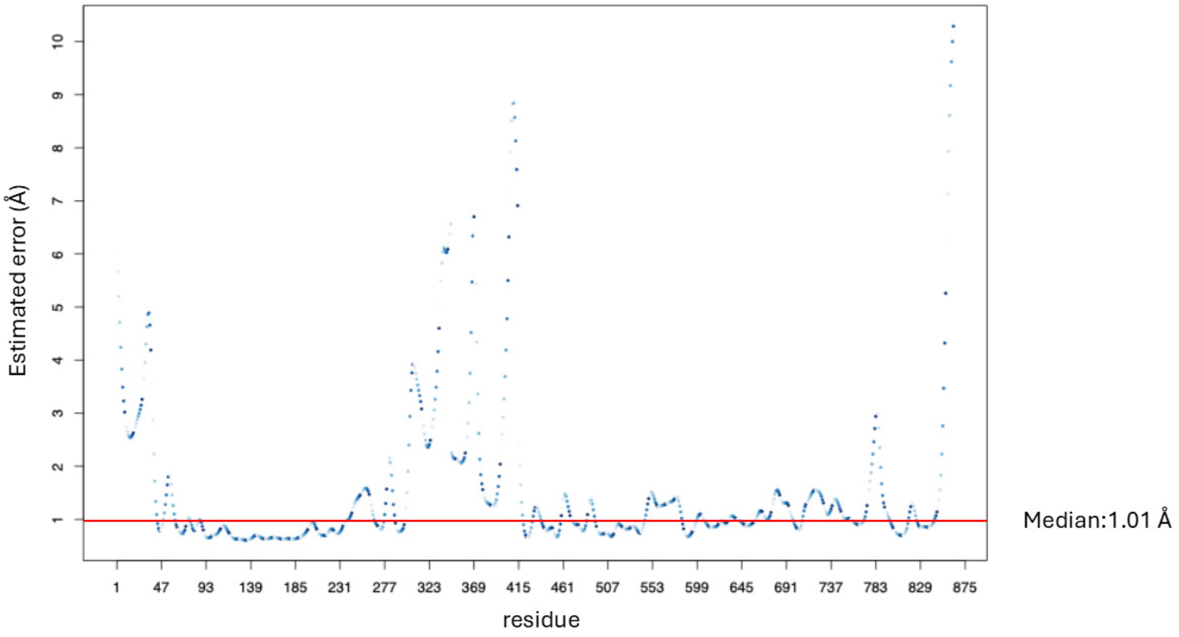

Figure S3. Error vs. Residue Plot. The model shows very low error for most residues, with the median error at 1.01 Å.

Supplementary methods.
P*seudomonas* ef1 species determination.
Mash's output shows, for each comparison between the *Pseudomonas* ef1 genome and the reference genome NZ_JAHSTX010000002 (which corresponds to *P. triticicola*), two key values:

- id:f:0.966939 → estimate of the average nucleotide similarity (≈96.7% identity),
- kc:f:1.00442 → fraction of the genome "covered" by the sketch (≈100% coverage).

Since the ANI estimated by Mash is ~96.7% and the coverage is nearly total, it exceeds the standard thresholds (ANI ≥95–96%, coverage ≥65%). These results suggest that *Pseudomonas* ef1 belongs to the same species as *Pseudomonas triticicola*.
